# A Randomized Controlled Trial: eHealth Interventions for Self-Management and Psychological Coping in Cancer Patients

**DOI:** 10.3390/healthcare14152391

**Published:** 2026-08-04

**Authors:** Manal Saleh Moustafa Saleh, Safaa Gaber Aly Salem, Khaled Abdallah Khader, Riham Hashem Fathi, Hanan E. Elsabahy, Hamad G. Dailah, Hind Mobark Fahd Alshamry, Shaimaa Mohamed Nageeb Mohamed, Eman Ahmed Gouda Ahmed, Laila A. Hamed

**Affiliations:** 1 Nursing Administration, Faculty of Nursing, Zagazig University, Zagazig 44519, Egypt; 2 Department of Nursing Sciences, College of Applied Medical Science, Shaqra University, Shaqra 11961, Saudi Arabia; 3 Department of Maternity, Obst & Gyn Nursing, College of Nursing, Princess Nourah bint Abdulrahman University, P.O. Box 84428, Riyadh 11671, Saudi Arabia; 4 Acute and Chronic Care Nursing Department, Faculty of Nursing, Al-Ahliyya Amman University, Amman 11118, Jordan; 5 Faculty of Medicine, Zagazig University, Zagazig 44519, Egypt; 6 Nursing Administration, Faculty of Nursing, Mansoura University, Mansoura 35511, Egypt; 7 College of Nursing and Health Sciences, Jazan University, Jazan 82817, Saudi Arabia; 8 Department of Medical Surgical Nursing, College of Nursing, University of Ha’il, Ha’il 55421, Saudi Arabia; hm.alshamry@uoh.edu.sa (H.M.F.A.);; 9 Psychiatric and Mental Health Nursing, Faculty of Nursing, Zagazig University, Zagazig 44511, Egypt; 10 Psychiatric and Mental Health Nursing, Faculty of Nursing, Hail University, Ha’il 55421, Saudi Arabia; 11 Obstetrics and Gynecology Nursing, Faculty of Nursing, Zagazig University, Zagazig 44511, Egypt; 12 Maternity Nursing, Maternal & Child Health Nursing Department, Faculty of Nursing, Northern Border University, Arar 91911, Saudi Arabia; 13 Department of Medical Surgical Nursing, Faculty of Nursing, Zagazig University, Zagazig 44511, Egypt

**Keywords:** self-management, coping strategies, cancer patients, eHealth interventions

## Abstract

**Highlights:**

**What are the main findings?**

- The eHealth intervention significantly improved cancer patients’ self-management practices, including problem-solving, symptom management, and emotional/interpersonal regulation.
- Significant improvements were observed in coping behaviors among the study group, with better affect-oriented and problem-oriented coping strategies compared with the control group.

**What are the implications of the main findings?**

- eHealth interventions can be integrated into oncology care to strengthen cancer patients’ coping abilities and self-management during treatment and survivorship.
- Demographic factors such as age, education, and income should be considered when designing digital health interventions to improve their accessibility and effectiveness.

**Abstract:**

**Background:** Enhancing self-management and coping strategies in cancer patients is critical to improving their quality of life and treatment outcomes. eHealth interventions, such as mobile apps, telehealth platforms, and online support tools, offer accessible, personalized, and cost-effective solutions that empower patients to actively manage their symptoms, adhere to treatment plans, and reduce psychological distress. These digital tools play a pivotal role in bridging care gaps, especially for individuals facing barriers to in-person support, underscoring their growing importance in modern oncology care. **Aim:** The aim of this study was to evaluate the efficacy of an eHealth intervention in enhancing self-management and coping strategies in cancer patients. **Methods:** This study was designed as a randomized controlled trial (RCT) and is reported in accordance with the CONSORT guidelines to ensure transparent and comprehensive reporting. A study and control group pre-test/post-test design was employed to investigate the impact of the intervention in the cities of Shaqra and Sajir, Riyadh, Saudi Arabia. A total of 212 participants were recruited using the Symptom-Management Self-Efficacy Scale (SMSE) and the Jaloweic Coping Scale. **Result:** Before the intervention, the correlation between coping behaviors and self-management practices was weak and statistically insignificant for both groups (study: r = 0.091, *p* = 0.351; control: r = 0.048, *p* = 0.622). After the intervention, a significant positive correlation emerged in the study group (r = 0.267, *p* = 0.006), suggesting that improvements in coping behaviors were associated with enhanced self-management practices. However, no significant change was observed in the control group (r = 0.081, *p* = 0.412). **Conclusions:** The intervention significantly improved self-management practices and coping behaviors in the study group, while the control group showed minimal or no improvement. The findings indicate that self-management and coping strategies are interrelated, and demographic factors such as age, education, and income influence the extent of improvement. **Trial Registration**: ClinicalTrials.gov identifier NCT07457112, registered on 5 March 2026, retrospectively registered.

## 1. Background

By 2030, there will likely be 28.7 million cancer survivors worldwide, 13.2 million cancer deaths, and 21.4 million new cases of cancer [1]. Given this escalating global burden, examining national cancer trends is crucial for understanding how local healthcare systems are being impacted. Recent national statistics indicate that Saudi Arabia is experiencing a parallel rise in cancer incidence. From 5616 cases in 2001 to 15,688 cases in 2018, the total number of cancer cases in Saudi Arabia increased by 179.3% for both sexes combined [2,3].

Due to the disease process and the burden of cancer treatment regimens, patients experience profound physical, psychological, and spiritual challenges. Many of these effects remain undertreated in clinical practice, contributing to increased morbidity and imposing a substantial burden on both patients and the healthcare system. To mitigate these consequences, patients and their families must adopt behaviors that promote recovery, support daily functioning, and reduce long-term risks. This highlights the importance of effective self-management practices that empower patients to take an active role in their care [4].

eHealth interventions have emerged as powerful tools in supporting patient self-management and coping during the cancer journey [5]. These digital platforms, ranging from mobile health applications to web-based educational and support systems, provide accessible, personalized resources that enable patients to monitor symptoms, adhere to treatment, receive real-time guidance, and reduce psychological distress. Recent evidence suggests that eHealth solutions enhance patient engagement, improve self-efficacy, and contribute to better clinical and psychological outcomes [6,7].

Throughout diagnosis, treatment, and survivorship, cancer patients face multiple physical, emotional, and psychological demands that disrupt daily routines and often diminish their sense of control. These challenges highlight the importance of strengthening both self-management and coping mechanisms, as the two processes are closely interrelated. Effective symptom control and emotional regulation rely on adaptive coping strategies such as problem-solving, seeking social support, and engaging in relaxation or spiritual practices, while self-management enables patients to understand their illness, make informed decisions, and maintain healthy behaviors. Research shows that interventions such as patient education, cognitive–behavioral therapy, support groups, and digital health technologies play a key role in promoting adaptive coping, improving psychological well-being, and enhancing overall quality of life [8,9].

Despite the global expansion of eHealth interventions targeting self-management and coping in cancer care, research within the Saudi context remains limited. Most available studies in Saudi Arabia focus on general cancer care or patient education, with very few examining the behavioral, psychological, and technological aspects of eHealth-based interventions. This scarcity of context-specific evidence highlights the need for more empirical research to determine the effectiveness of digital health tools in enhancing coping skills and self-management among Saudi cancer patients.

## 2. Theoretical Framework

A relevant theoretical framework for understanding the effectiveness of eHealth interventions in enhancing self-management and coping strategies among cancer patients is the Self-Determination Theory (SDT). SDT posits that individuals are more likely to engage in and sustain health behaviors when their basic psychological needs for autonomy, competence, and relatedness are supported. eHealth tools, such as mobile applications and online platforms, can foster autonomy by allowing patients to monitor symptoms, set goals, and access tailored information at their own pace. These technologies also enhance competence by providing feedback and educational resources that build confidence in managing illness-related challenges. Additionally, forums and virtual support groups can fulfill relatedness needs by connecting patients with peers and healthcare professionals. Thus, SDT provides a strong conceptual basis for the design and evaluation of eHealth interventions aimed at empowering cancer patients in their self-care journey [10].

### 2.1. Effect of Role of eHealth Interventions on Enhancing Self-Management in Cancer Patients

Several studies have demonstrated the significant role of eHealth interventions in enhancing self-management among cancer patients. For example, a mixed-methods study by [11] found that an interactive mobile application for symptom self-management improved patients’ engagement, symptom monitoring, and overall quality of life during breast and prostate cancer treatment. Systematic reviews have similarly reported that digital self-management support tools, including tailored feedback and interactive features, effectively reduce symptom burden and improve treatment adherence [12,13]. Moreover, web-based interventions and digital health coaching programs have been shown to enhance patients’ ability to manage fatigue, emotional distress, and anxiety during chemotherapy, while real-time symptom tracking enables timely clinical interventions, potentially reducing emergency visits [12,14]. Collectively, these findings underscore that eHealth interventions play a crucial role in supporting cancer patients’ self-management capabilities throughout the care continuum.

### 2.2. Effect of the Role of eHealth Interventions on Coping Strategies in Cancer Patients

Several recent studies have investigated the role of eHealth interventions in enhancing coping strategies among cancer patients, showing promising results in improving psychological and emotional well-being. Ref. [15] found that interactive digital tools designed for cancer patients supported empowerment, increased engagement in self-care, and promoted adaptive coping strategies. Similarly, ref. [16] reported that web-based self-management interventions effectively enhanced problem-focused coping and reduced distress in cancer patients. Mindfulness-based eHealth interventions have also shown beneficial effects; Ref. [17] demonstrated that these interventions reduced cancer-related symptoms and improved adaptive coping among patients and survivors. Consistent with these findings, ref. [18] reported that online mindfulness programs improved emotional regulation, reduced anxiety, and enhanced coping mechanisms in cancer patients. Additionally, ref. [19] highlighted that tailored eHealth interventions for breast cancer patients increased engagement with self-care behaviors and improved resilience, indirectly supporting coping strategies. These findings collectively suggest that eHealth interventions are valuable tools in promoting adaptive coping and enhancing quality of life in cancer care.

### 2.3. Effect of Enhancing Self-Management and Coping Strategies in Cancer Patients Through the Role of eHealth Interventions

The benefits of eHealth interventions in cancer care have been increasingly emphasized in recent studies. For example, web-based and smartphone-based interventions have been shown in systematic reviews and meta-analyses to improve quality of life, lower symptoms of anxiety and depression, and increase self-efficacy in cancer patients [20,21,22]. Similarly, among cancer patients, app-based therapies have shown positive impacts on psychological outcomes like anxiety and depression as well as on fatigue, sleep quality, and pain management [23]. Additionally, web-based therapies for breast cancer survivors have demonstrated modest but noteworthy increases in quality of life, especially in the areas of emotional, cognitive, and physical functioning [24]. Together, these results bolster the mounting evidence that eHealth technologies, such as web programs and mobile apps, can significantly improve cancer patients’ and survivors’ psychological well-being, coping skills, and self-management.

### 2.4. Significance of the Study

The significance of this study lies in its focus on improving the quality of life and health outcomes for cancer patients by enhancing their self-management and coping strategies through eHealth interventions. As cancer care increasingly shifts toward outpatient settings, patients are often required to manage complex symptoms, treatment side effects, and emotional distress on their own. Despite the growing availability of eHealth tools, the literature remains limited on their effectiveness in fostering sustainable self-management behaviors and coping mechanisms tailored to cancer patients. Most existing studies have either focused on general chronic conditions or offered limited evaluations of patient-centered digital tools. This study addresses this gap by examining how well-designed eHealth interventions can empower cancer patients in their daily lives, providing accessible, personalized, and scalable support to help them manage their condition more effectively.

### 2.5. The Aim of the Study

#### 2.5.1. Aim

This research aimed to evaluate the efficacy of eHealth interventions in enhancing self-management and coping strategies in cancer patients.

#### 2.5.2. Study Objectives

(1) To assess cancer patients’ level of self-management before and after intervention.
(2) To examine cancer patients’ ability to cope with cancer before and after intervention.
(3) Designing and implementing an educational and training intervention to promote self-management and healthy coping behaviors among cancer patients.
(4) To evaluate the effect of cancer patients’ self-management and coping behaviors before and after implementing the proposed educational interventions.

## 3. Methods

### 3.1. Study Design, Setting, and Participants

This study employed a randomized controlled trial (RCT) to evaluate the effectiveness of an eHealth intervention on self-management practices and coping behaviors among patients with cancer in Shaqra and Sajir cities, Riyadh Province, Saudi Arabia. The trial was conducted and reported in accordance with the CONSORT guidelines to ensure transparent and comprehensive reporting. A two-group, pretest–posttest design was used, allowing comparisons of outcomes both within groups (before and after the intervention) and between the study and control groups.

**Figure 1:**
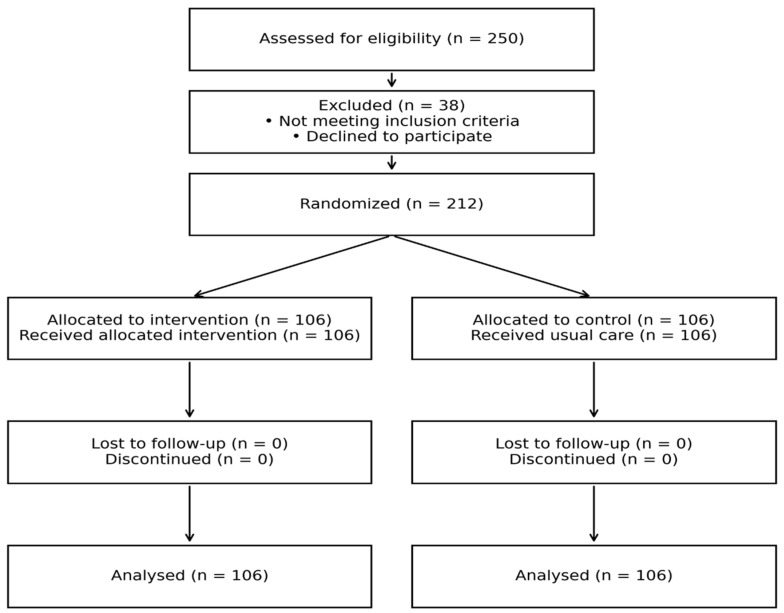
CONSORT Flow Diagram of participant recruitment, allocation, follow-up, and analysis.

Eligible participants were adult patients diagnosed with cancer who were either undergoing treatment or had completed treatment (chemotherapy, radiotherapy, surgery, or a combination of these modalities), were able to read and understand Arabic, and were willing to participate in the study. Patients with learning disabilities, those receiving psychiatric treatment, those requiring additional social support services, or those unable to perform activities of daily living independently were excluded. A total of 212 participants were enrolled and randomly allocated to either the intervention group (n = 106) or the control group (n = 106), see Figure 1.

### 3.2. Sample Size Calculation

A total of 106 cancer patients were enrolled in each group (study and control) based on an a priori sample size calculation. The calculation used the formula: n = Z^2^_{1 − \alpha/2} × p(1 − p)/d^2^, where Z_{1 − \alpha/2} = 1.96 corresponds to a 5% type I error (*p* < 0.05), p is the expected proportion from previous studies, and d = 0.05 represents the margin of error (5%). Participants were then randomly assigned to the study and control groups using a computer-generated randomization list, ensuring comparable baseline characteristics. Any missing data were handled through intention-to-treat analysis, and potential confounders were controlled during statistical analysis.

### 3.3. Randomization and Blinding

The “Research Randomizer,” an online application [25], was used by an independent researcher to randomly allocate participants to study or control groups. The algorithm created two sets of 106 distinct, sorted numbers after the number of groups and participants was specified. These were then randomly assigned in a 1:1 ratio to either Group 1 (the study group) or Group 2 (the control group). The opaque sealed envelopes were used to hide the allocation order. It was not possible to blind the researcher or participants to group assignments because the intervention was an instructional program [26]. However, to minimize bias, the authors independently collected and analyzed the data without participating in the intervention program.

### 3.4. Reliability and Validity of Instruments

Seven professors and assistant professors from the university’s nursing academic staff examined the translated instruments after the Symptom-Management Self-Efficacy Scale and the Jaloweic Coping Scale were translated into Arabic utilizing [27] translation methodology. The panel also examined the face and content validity of the measurement tools for the Arabic-to-English back translation. Based on the panel’s suggestions, nothing needed to be changed. The instrument’s internal consistency was assessed using reliability analysis. To assess the measurement reliability, Cronbach’s alpha coefficients were computed for multipoint items.

The researcher assessed reliability to evaluate the internal consistency of the instruments; these instruments were tested for reliability to estimate measurement consistency. Reliability was assessed using the alpha coefficient (Cronbach’s alpha). Across all data collection tools, the Factor loadings were acceptable, assessed, and confirmed to be above 0.40, supporting the questionnaire’s dimensional structure.

### 3.5. Procedures

After obtaining formal authorization, the researchers began recruiting patients who met the inclusion criteria. Participants were approached individually to obtain oral consent, and WhatsApp groups were used to facilitate communication and cooperation. The study consisted of three main phases: recruitment and interviewing, intervention implementation, and evaluation.

#### 3.5.1. Recruiting and Interviewing

Patient recruitment occurred between November 2023 and February 2024. The study purpose, intervention details, and one-month and three-month requirements were explained to all potential participants. Recruited patients were divided into seven groups, with 13–17 participants per group, and a WhatsApp group named ‘Empowering Healthy Coping Behaviors’ was created for each.

Before pre-test data collection, the researchers sent messages twice daily in the WhatsApp groups to foster rapport and encourage peer communication. The WhatsApp platform was used to provide information about effective self-management and coping strategies.

#### 3.5.2. Implementation

Participants completed a structured electronic questionnaire via Google Forms. A link was distributed via WhatsApp and email, and participants confirmed consent by selecting “Yes, I agree to participate” before proceeding. Pre-tests were completed before the educational sessions.

The educational program lasted six weeks, with two sessions per week (30 h in total). Sessions were delivered via Google Meet and Zoom and included lectures, group discussions, and scenario-based learning. Teaching materials, including posters, videos, and pamphlets, were shared through smartphone support services. Session content was developed based on an extensive literature review, assessment results, and identified gaps in patients’ self-management and coping skills. Monitoring fidelity and adherence: Attendance was recorded for each online session. Monitoring fidelity and adherence was ensured by recording attendance for each session and tracking participants’ engagement on WhatsApp (e.g., reading messages and responding). Participants received reminders twice weekly to complete assignments and review educational materials, supporting consistent participation throughout the program.

#### 3.5.3. Evaluation Phase

A post-test was conducted immediately after the intervention to evaluate its effectiveness in improving self-management and coping behaviors among cancer patients. Changes in participants’ knowledge and practices were analyzed to assess the intervention’s impact.

No harms or unintended effects were observed or reported in either group, indicating that the intervention was safe and well-tolerated.

Control Group. Participants in the control group received only the materials from the first and second sessions. They did not participate in e-health activities, reflection sessions, or the remaining three-week intervention.

Table 1 summarizes the intervention activities over six weeks, delivered through two online sessions per week. The content progresses from basic self-management and coping strategies to advanced discussions, followed by review and reinforcement, while adherence is monitored through attendance, WhatsApp engagement, and assignment completion.

### 3.6. Data Collection Tools

#### 3.6.1. The Symptom-Management Self-Efficacy Scale (SMSE)

Firstly, collected data on participants’ health profiles, including their medical condition (i.e., cancer), such as diagnosis, disease duration, medications used, treatment-related side effects, and comorbidities and/or complaints.

Secondly, the symptom-management self-efficacy scale was developed by [28] to evaluate the key behaviors and tasks related to symptom management during chemotherapy. In total, 27 items are included in the scale, representing three subscales: acquiring problem-solving (AP, seven items), managing chemotherapy-related symptoms (MC, 15 items), and managing emotional and interpersonal disturbance (ME). Furthermore, the SMSE scale has 11 points, with 0 meaning that a person is not at all confident and 10 meaning that they are completely confident in a variety of actions. The total score ranges higher, reflecting stronger perceived self-efficacy in symptom management. According to [28], the internal reliability for the 27-item SMSES scale was 0.96 with a Cronbach alpha range of 0.88 to 0.95 for all subscales of the measurement tool.

#### 3.6.2. The Jaloweic Coping Scale

The Jaloweic Coping Scale was developed in 1981 by Jaloweic and Powers. The scale was used to determine how older patients with various chronic conditions cope with their diseases. The scale can be utilized to evaluate coping behaviors in common circumstances as well as with certain conditions [29,30]. The Jaloweic scale consists of 40 items representing two subscales. The first subscale, affective-oriented behaviors (25 items), is intended to handle distressing emotions such as worry, wanting to be alone, and working off tension with physical activity. The second subscale, problem-oriented coping behaviors (15 items), focuses on problem resolution as looking at the problem objectively and accepting the situation as it is. Furthermore, the scale items are answered using a five-point Likert scale as follows: 0 = “never”, 1 = “occasionally”, 2 = “roughly half the time”, 3 = “frequently”, and 4 = “always.” An alpha of 0.86 and a test–retest reliability of 0.79 were reported, which shows good estimates for the scale [29,30]. In addition, a higher score suggests greater use of that specific coping strategy [29].

#### 3.6.3. Demographic Information Form

Participants’ age, gender, place of residence, marital status, educational attainment, occupational status (whether current or retired), and income.

### 3.7. Data Collection and Procedure

Data were collected from male and female patients diagnosed with cancer who met the eligibility criteria and agreed to participate in the study. Participants were recruited from hospitals in Shaqra and Sajir cities, Riyadh Province, Saudi Arabia, between November 2023 and February 2024. Following baseline assessment, participants were randomly allocated to either the study or the control group using an online randomization procedure. The intervention was delivered in seven groups, each comprising 13–17 participants.

The eHealth educational program was delivered through mobile phones to facilitate continuous communication and access to educational materials. The intervention was implemented under the supervision of the chief nurses and incorporated a variety of teaching strategies, including interactive lectures, group discussions, brainstorming sessions, role-playing activities, small-group work, and educational video materials. Baseline and post-intervention assessments were completed using the study questionnaires according to the study protocol.

### 3.8. Pilot Study

Additionally, to evaluate the viability of the study’s instruments as well as their clarity, applicability, and time needs, a preliminary pilot study including ten male and female cancer patients was carried out. Following the integration of the pilot study’s findings, the study’s conclusions stayed the same.

### 3.9. Ethical Considerations

Ethical approval for this study was obtained from the Standing Committee of Research Ethics at Shaqra University, Saudi Arabia (Approval No. ERC_SU_20230011). The study was conducted in accordance with the ethical principles of the Declaration of Helsinki.

Before participation, all eligible participants received detailed information regarding the study objectives, procedures, potential benefits, and their rights as research participants. Written informed consent was obtained from all participants before enrollment. For participants completing the electronic questionnaire, consent was confirmed electronically by selecting the statement, “Yes, I agree to participate,” before accessing the questionnaire.

Participation was entirely voluntary. Participants were informed of their right to withdraw from the study at any time without penalty or consequences. All collected data were treated confidentially, anonymized before analysis, and used exclusively for research purposes.

### 3.10. Data Analysis

Statistical analyses were performed using IBM SPSS Statistics version 20 (IBM Corp., Armonk, NY, USA). SPSS was selected because it provides validated and widely accepted procedures for descriptive and inferential statistical analyses, including normality testing, *t*-tests, correlation analysis, and analysis of variance (ANOVA), which are appropriate for the objectives and design of the present randomized controlled trial. Furthermore, SPSS is extensively used in clinical and nursing research, facilitating transparency, reproducibility, and comparison with previous studies.

Descriptive statistics were used to summarize participants’ demographic characteristics and study outcomes. Continuous variables were presented as means and standard deviations (SD), whereas categorical variables were expressed as frequencies and percentages. The normality of continuous variables was assessed using the Kolmogorov–Smirnov test.

Baseline demographic characteristics between the study and control groups were compared using the Chi-square test or Fisher’s exact test for categorical variables, as appropriate. Independent-samples *t*-tests were used to compare continuous variables between the two groups at baseline and post-intervention.

Changes in self-management practices and coping behavior scores before and after the intervention within each group were evaluated using paired-samples *t*-tests. Because the study included only two measurement occasions (pre-intervention and post-intervention), paired-samples *t*-tests were selected to assess within-group changes over time, while independent-samples *t*-tests were used to compare the study and control groups at each assessment point. This analytical approach was chosen to directly address the predefined study objectives of evaluating changes within each group following the intervention and differences between groups at baseline and post-intervention, providing a straightforward and clinically meaningful interpretation of intervention-related effects in a two-group, two-time-point study design.

Pearson’s correlation coefficient was used to examine the relationship between coping behaviors and self-management practices before and after the intervention in both groups. One-way analysis of variance (ANOVA) was performed to examine differences in coping behavior and self-management scores according to participants’ demographic characteristics. When statistically significant differences were identified, Least Significant Difference (LSD) post hoc tests were conducted for pairwise comparisons. All statistical tests were two-tailed, and statistical significance was established at a *p*-value of less than 0.05.

## 4. Results

Table 2: This table presents the demographic characteristics of the study (n = 106) and control (n = 106) groups. No significant differences were found between the groups in terms of age distribution (*p* = 0.985), gender (*p* = 0.846), marital status (*p* = 0.679), education level (*p* = 0.767), occupation (*p* = 0.266), income (*p* = 0.121), income source (*p* = 0.197), and living status (*p* = 0.464). The similarity in these characteristics ensures that differences in study outcomes are likely attributable to the intervention rather than pre-existing demographic disparities.

Table 3: The distribution of cancer types was similar between groups, with the most prevalent type in the study and control group being breast cancer (34.0% and 19.8%), while the 2nd cancer type in the study group was other 20.8%, and colon and blood cancer had a higher prevalence in the control group (18.9% and 17.9%), respectively. Cervical cancer (study: 8.5%, control: 10.4%) and prostate cancer (study: 13.2%, control: 16.0%) had comparable distributions. No statistically significant differences were observed in cancer type (*p* = 0.115) or stage (*p* = 0.255), indicating that disease severity was balanced between groups.

Table 4: This table reveals that significant improvements in self-management practices were observed in the study group following the intervention. Participants demonstrated notable progress in acquiring problem-solving skills, with scores increasing from 35.3 ± 15.8 to 41.0 ± 18.0 (*p* = 0.015). Similarly, their ability to manage chemotherapy-related symptoms improved substantially, rising from 61.9 ± 25.8 to 74.3 ± 32.5 (*p* = 0.002). Additionally, there was a significant enhancement in managing emotional and interpersonal disturbances, with scores increasing from 21.1 ± 10.5 to 24.3 ± 11.2 (*p* = 0.033). The total self-management score also showed a marked improvement, increasing from 118.2 ± 50.6 to 139.7 ± 59.3 (*p* = 0.005). In contrast, the control group exhibited minimal changes across all domains, with no statistically significant improvements recorded. These findings indicate that the intervention effectively enhanced participants’ self-management skills, equipping them with better strategies to navigate their condition.

Table 5: This table revealed that coping behaviors showed significant improvement in the study group following the intervention. Affective-oriented behaviors increased from 40.4 ± 14.1 to 44.5 ± 12.4 (*p* = 0.025), reflecting enhanced emotional regulation among participants. Similarly, problem-oriented coping behaviors improved from 25.7 ± 10.1 to 29.4 ± 9.7 (*p* = 0.007), indicating a greater ability to address challenges through practical strategies. The total Jaloweic coping score also showed a notable rise, increasing from 66.1 ± 21.2 to 73.9 ± 19.3 (*p* = 0.005), suggesting better coping mechanisms. In contrast, the control group exhibited no significant changes in coping behaviors. These findings highlight the positive impact of the intervention in strengthening both emotional and problem-solving coping strategies among participants.

**Figure 2:**
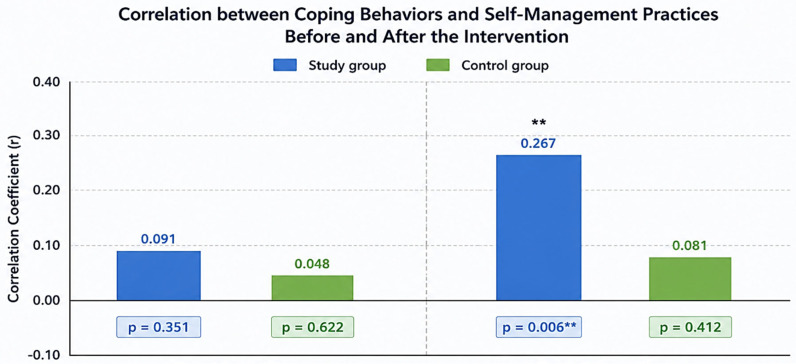
Correlation between Coping Behaviors and Self-Management Practices Scores ** *p* < 0.01 (statistically significant).

Figure 2 and Table 6 Correlation between Coping Behaviors and Self-Management Practices Scores. This Figure and table shows that before the intervention, the correlation between coping behaviors and self-management practices was weak and statistically insignificant for both groups (study: r = 0.091, *p* = 0.351; control: r = 0.048, *p* = 0.622). After the intervention, a significant positive correlation emerged in the study group (r = 0.267, *p* = 0.006), suggesting that improvements in coping behaviors were associated with enhanced self-management practices. However, no significant change was observed in the control group (r = 0.081, *p* = 0.412).

Table 7A,B: Association Between Demographic Characteristics and Coping Behaviors’ Mean Scores: This table show that post-intervention analysis revealed significant associations between demographic characteristics and coping behaviors in the study group. Participants with higher education levels demonstrated greater improvement, with university-educated individuals achieving a mean score of 81.2 ± 24.2 compared to 72.7 ± 24.8 among those with secondary education (*p* = 0.032). Similarly, income level played a crucial role in coping behavior outcomes. Participants with “not enough” income had the lowest post-intervention scores (60.6 ± 23.7), whereas those with “enough” income showed higher scores (74.1 ± 24.9, *p* = 0.026), and those with “enough and more” income exhibited the greatest improvement (80.8 ± 21.1, *p* = 0.028). These findings suggest that both education and financial stability may influence individuals’ ability to develop effective coping strategies. In contrast, no significant associations between demographic factors and coping behaviors were observed in the control group, indicating that the intervention played a key role in these improvements.

Table 8A,B: Association Between Demographic Characteristics and Mean Scores of Self-Management Practices. Post-intervention analysis revealed significant improvements in self-management practices among the study group, influenced by various demographic factors. Age played a crucial role, with participants over 40 years demonstrating the greatest improvement, reaching a mean score of 168.6 ± 51.7 compared to 150.4 ± 58.3 among those aged 30–40 (*p* < 0.001). Marital status also had a significant impact, as married participants exhibited greater progress (163.6 ± 51.9) compared to single participants (111.6 ± 56.7, *p* < 0.001). Additionally, education level was a key determinant, with university-educated individuals achieving significantly higher self-management scores (166.9 ± 61.4) than those with secondary education (124.4 ± 53.2, *p* = 0.004). Income level further influenced outcomes, with participants having “enough and more” income showing the greatest improvement (165.0 ± 57.2, *p* = 0.014). In contrast, the control group exhibited no statistically significant associations between demographic factors and self-management practices, highlighting the effectiveness of the intervention in enhancing self-management skills among specific demographic groups.

## 5. Discussion

Enhancing cancer patients’ coping strategies and self-management through eHealth interventions has become an important area for research and application. By providing patients with tools to monitor their health, access information, and communicate with medical professionals, these digital resources help patients feel more empowered and in control of their treatment journey. According to a systematic review, interactive digital tools significantly boost patient empowerment, self-management, and coping strategies, showing positive results in areas like emotional well-being and self-efficacy [15]. Moreover, the development of specific eHealth platforms, such as the I Manage Cancer project, underscores the importance of integrating psycho-emotional support and family resilience into these interventions, addressing the needs of both patients and caregivers at each stage of the cancer journey [31,32]. As cancer care continues to advance, utilizing eHealth interventions will be vital in improving the quality of life for cancer patients and their families.

Regarded to distribution of Participants Based on Their Cancer Type. The most prevalent type in the study and control groups was breast cancer. This result may be related to Breast cancer, which is influenced by a combination of genetic, hormonal, and environmental factors. Genetic mutations, particularly in the BRCA1 and BRCA2 genes, significantly increase the risk. Hormonal factors, such as prolonged exposure to estrogen, can also play a role, which may be linked to factors like early menstruation, late menopause, and the use of hormone replacement therapy. Additionally, lifestyle choices, including obesity, alcohol consumption, and lack of physical activity, contribute to the risk. Environmental exposures, such as radiation and certain chemicals, further underscore the complex interplay of factors that may lead to the development of breast cancer. This result agrees with [33], according to Cancer statistics 2025, which reported that the incidence continues to increase for six of the top 10 cancers (breast, prostate, melanoma, uterine corpus, pancreas, and CRC [aged <65 years]), two of which primarily affect women. Consequently, the cancer burden is shifting from older to younger adults and from men to women. Middle-aged women now have a slightly higher cancer risk than their male counterparts, and young women are almost twice as likely to be diagnosed as young men. Ref. [34] agree with these study results and added that Patients with early and advanced BC desire psychological support, concise educational resources, and holistic care. Ref. [35] who concluded that the increasing incidence of BC among WCBA highlights the urgent need for effective intervention and preventive policies to alleviate this growing global burden. Ref. [36] monitoring the effectiveness of continuing efforts to manage breast cancer, such as the World Health Organization Global Breast Cancer Initiative, which seeks to reduce breast cancer mortality by 2.5% annually, depends on updates of current and expected estimates of the burden.

The current study showed that the eHealth-based intervention significantly improved the coping strategies and self-management techniques of cancer patients. These results are consistent with Self-Determination Theory (SDT), which holds that improving relatedness, competence, and autonomy promotes long-term behavioral change. In this study, the digital intervention strengthened relatedness through ongoing digital communication and emotional support, which helped participants feel connected to healthcare providers throughout their treatment journey; competence was reinforced through structured educational materials that increased patients’ confidence in managing treatment-related challenges; and autonomy was fostered by enabling participants to independently monitor symptoms and access health information. The observed gains in coping and self-management can be theoretically explained by integrating these three SDT constructs.

The results indicate enhanced coping behaviors, both affective-oriented (emotional regulation) and problem-oriented (practical coping strategies), following the intervention. These improvements are consistent with prior literature showing that coping self-efficacy (CSE) plays a critical role in managing cancer-related stress. For example, in patients undergoing lung cancer treatment, higher self-care self-efficacy and positive coping mediated the relationship between social support and psychological resilience, resulting in better mental health outcomes [37]. Similarly, greater self-efficacy for managing cancer has been associated with lower distress and better perceived general health [38]. This supports the reviewer’s point that connecting findings to theoretical frameworks, in this case SDT, provides deeper insight into the mechanisms by which interventions improve coping and self-efficacy.

The observed improvements in self-management practices, including enhanced problem-solving, symptom management, and emotional/interpersonal regulation, are consistent with prior evidence linking self-efficacy to self-management behaviors in cancer patients. An integrative review highlighted that self-management behaviors span multiple domains (symptom management, emotional management, lifestyle, follow-up adherence) and are influenced by psychological factors, personal factors, social support, and system-level aspects [39]. Moreover, a cross-sectional study from Saudi Arabia showed that higher self-efficacy predicts better self-management among adults undergoing cancer treatment [40]. Responding to the reviewer’s comment about contextual factors and limitations, we also acknowledge that cultural attitudes, digital literacy, and resource constraints in Saudi Arabia may affect intervention scalability, and future interventions should consider these barriers for broader applicability.

This is similar to the result reported by [41] of low self-management efficacy in patients with high levels of fear of pain. When faced with negative emotions that arise during the treatment of the disease, patients with high self-management efficacy believe that they will be able to overcome the disease and externalize this belief into action by actively seeking medical treatment. Ref. [42] showed that patients with high self-management efficacy were more likely to express their worries and fears to healthcare workers, which was effective in relieving psychological stress. Even if cancer recurs, confiding could help patients cope calmly, thereby reducing the fear of recurrence. It is recommended that medical staff carry out psychological counseling interventions for inpatients with breast cancer to stimulate patients’ potential beliefs, help them improve their self-management efficacy, and reduce their fear of recurrence.

Results related to Participants’ Coping Behaviors. showed significant improvement in the study group following the intervention. Jaloweic Coping was the highest in the two-group but improved in the study group more than the control group following affective and problem-oriented coping Behaviors. This result may be related to the effectiveness of the intervention in enhancing participants’ coping mechanisms by providing them with structured strategies and emotional support. The significant improvement in both affective-oriented and problem-oriented coping behaviors suggests that the intervention not only helped individuals manage their emotional responses more effectively but also equipped them with practical tools to tackle stressors. The rise in the total Jalowiec coping score further supports the conclusion that the intervention fostered comprehensive coping improvements, while the lack of significant change in the control group reinforces the intervention’s specific impact.

Several recent studies support the current findings, highlighting the effectiveness of targeted interventions in enhancing coping strategies. For instance, ref. [43] reported significant improvements in emotional regulation and problem-solving abilities among nursing students following participation in a resilience training program. The intervention group exhibited a notable increase in both affective- and problem-oriented coping behaviors, as reflected by higher total scores on the Jalowiec Coping Scale (JCS), whereas the control group showed no comparable change. These results are consistent with previous research. Ref. [44] found that training in problem-solving skills significantly increased problem-focused coping, reduced emotion-focused coping, and enhanced emotional self-efficacy among participants compared to controls. Similarly, ref. [45] demonstrated that a structured stress management program significantly improved coping strategies and self-efficacy relative to the control group. Collectively, these studies reinforce the conclusion that interventions providing structured coping skills training and emotional support can effectively promote emotion regulation and proactive problem-solving, leading to more flexible and adaptive coping among participants.

Despite the positive outcomes reported in many intervention studies, not all research confirms that coping-skills programs lead to durable or comprehensive improvements. For instance, a meta-analysis of interventions aimed at improving coping strategies in older adults found a significant enhancement only in problem-focused coping (SMD = 0.37, *p* = 0.005), while there was no significant effect on emotion-focused coping [46]. Moreover, a systematic review of stress-management and behavior-change interventions among student nurses concluded that although some interventions (e.g., mindfulness, relaxation) may yield short-term stress reduction, the evidence remains insufficient to determine which methods reliably foster long-term coping improvements [47]. Similarly, perceived stress, stressors, and coping strategies among nursing students in the Middle East and North Africa showed mixed results, indicating that improvements in one coping domain may not generalize across all styles [48]. These findings suggest that the effectiveness of coping-skills interventions may be limited by factors such as the target population, the coping domains studied (problem- vs. emotion-focused), and the sustainability of effects; hence, improvements observed in one domain or shortly after intervention may not generalize across all coping styles or persist over time. Overall, the literature underscores the necessity for tailored interventions that enhance adaptive coping strategies while addressing the potential pitfalls of maladaptive behaviors, as emphasized in recent studies [49].

Correlation between Coping Behaviors and Self-Management Practices. The findings of the present study showed that after the intervention, a significant positive correlation emerged in the study group (r = 0.267, *p* = 0.006), suggesting that improvements in coping behaviors were associated with enhanced self-management practices. However, no significant change was observed in the control group. This result is in the same line with [50]. The study used Mastery Enhancement Therapy as an intervention for cancer patients, showing significant gains in coping self-efficacy and depression reduction. The intervention was time-effective, optimized coping behavior, and improved compliance, with small drop-out rates. It was particularly effective for advanced cancer patients with more symptoms when compared to a control group. Also, ref. [31] agree with the result and reported that Self-management interventions showed promise for improving QoL, but study quality was variable, with substantial heterogeneity in intervention characteristics and components. By identifying what to adapt from existing interventions, these findings can inform the development and implementation of self-management interventions in cancer. Moreover, ref. [51] agree with the study result and added that the study reveals that cancer survivors use self-management strategies to care for their health and well-being, including social networks, healthy behaviors, motivation, objective decision-making, and creating favorable environments. These strategies aid in cancer treatment rehabilitation, current condition management, and future health protection.

Similarly, study [52] found that coping styles and self-care acted as separate and sequential mediators in the relationship between stigma and post-traumatic growth among patients who underwent enterostomy. Therefore, interventional strategies targeting coping styles and self-care may provide synergistic benefits to patients with permanent enterotomy by enhancing their post-traumatic growth.

Participants’ Demographic Characteristics and Coping Behaviors of the present study showed that post-intervention analysis revealed significant associations between demographic characteristics and coping behaviors in the study group. This suggests that various demographic factors, such as age, gender, socioeconomic status, and education, can influence how cancer patients cope with their diagnosis and treatment, highlighting the importance of personalized support and interventions based on these characteristics.

Participants with higher education levels demonstrated greater improvement, compared to university-educated individuals. Similarly, income level played a crucial role in coping behavior outcomes. Participants with “not enough” income had the lowest post-intervention scores, whereas those with “enough” income showed higher scores, and those with “enough and more” income exhibited the greatest improvement. These findings suggest that both education and financial stability may influence individuals’ ability to develop effective coping strategies. In contrast, no significant associations between demographic factors and coping behaviors were observed in the control group, indicating that the intervention played a key role in these improvements.

Research indicates that demographic factors, including education and income, significantly impact how cancer patients cope with their diagnosis and treatment [53,54]. Higher education levels are often associated with better coping strategies and psychological resilience, which can lead to improved mental health outcomes. Income levels can affect access to healthcare resources, support systems, and coping mechanisms. Patients with higher incomes may have better access to psychological support and treatment options, which can enhance their coping abilities. The literature supports the notion that effective coping behaviors are crucial for psychological adjustment in cancer patients. Those who employ adaptive coping strategies tend to experience less distress and better overall well-being. While the study suggests significant associations, it is essential to recognize that coping responses can vary widely among individuals, regardless of demographic factors. According to a recent multicenter study, psychological and lifestyle factors (self-efficacy, sense of coherence, physical activity) had stronger correlations with resilience than demographic variables (education, income), indicating that coping outcomes are not entirely determined by demographic factors alone [55].

The analysis of demographic factors revealed notable variations in intervention outcomes. Higher education, adequate income, older age, and marital status were associated with greater improvements in coping and self-management practices. These findings reflect the influence of socioeconomic resources and health literacy on patients’ capacity to adopt and sustain health behaviors, as supported by prior studies in Saudi Arabia and internationally [40]. However, Higher wealth and education do not always guarantee the best self-management practices among patients with chronic diseases, according to recent research. Rather, self-efficacy, social support, and psychological preparedness are crucial in determining health-related behaviors. For example, ref. [56] used a path analysis to show that, while socioeconomic determinants had a reduced direct effect, social support and self-efficacy significantly led to effective self-management practices among Saudi individuals with chronic disease. These results highlight the significance of using tailored approaches that boost patients’ self-esteem and make use of their support systems instead of depending only on demographic indicators of health behaviors.

Despite the positive outcomes, several contextual and methodological limitations should be acknowledged. First, the digital nature of the intervention may limit its accessibility among patients with low digital literacy, older adults unfamiliar with technology, or individuals lacking adequate internet access, factors particularly relevant in certain regions of Saudi Arabia. Second, cultural attitudes regarding help-seeking, emotional expression, and reliance on family may influence how patients engage with coping and self-management strategies. Third, the intervention’s scalability may be challenged by variability in patients’ socioeconomic backgrounds, differences in healthcare infrastructure across settings, and the need for ongoing technical support to maintain program fidelity. Additionally, although the intervention improved autonomy and competence, some participants may still require more intensive, face-to-face psychological or medical support.

Also, ref. [57] this study provides important understandings into the relationship between self-management skills (SMS) and demographic factors (DF) among Kathmandu-based organizations in Nepal. According to the findings, gender, age, and training are important predictors of SMS levels; however, SMS mean was not correlated with educational level, married status, or SMS training. Additional study displays that Self- Management Skills has an encouraging impact on performance, supporting positive emotions, adaptableness, empathy, and ethical behavior. The research study highlights that age, gender, and training are all positively related to SMS, actually enhancing their self-esteem, SMA, and AA. By focusing on these critical components, organizations can adopt effective services and SMS to increase their general performance. However, some authors have expressed disagreement with these findings, suggesting that the relationship between demographic factors and self-management practices may not be as straightforward as indicated.

Although our results differ in certain aspects, they remain consistent with the evidence reported by [41], who found that fear of disease progression (FoP) was significantly higher among younger breast cancer patients and was strongly associated with lower self-management efficacy and poorer family functioning. Their regression analysis explained 36% of the variance in FoP, underscoring the inverse relationship between FoP and self-management capability. These findings reinforce the validity of our exploratory prediction model, as our data similarly indicate that reduced self-management efficacy is linked to heightened FoP, suggesting that patients with lower self-regulatory capacity may be at greater risk of experiencing elevated FoP.

There was a statistically significant improvement in knowledge and self-management in the study group compared with the control group after implementation of an education program based on the common-sense model of self-regulation. This result agrees with a randomized controlled trial [58,59] that found that a CSM-based education program significantly improved self-management scores in patients compared to controls. Also, Results from a recent Egyptian clinical study named “Effect of Education Program-Based on Common Sense Model of Self-Regulation on Self-Management for Patients with Helicobacter Pylori Infection” confirm the current conclusion. After the CSM-based educational program was implemented, the researchers found that the study group’s knowledge and self-management improved significantly when compared to the control group. This demonstrates that structured therapies based on the Common-Sense Model successfully improve patients’ comprehension of their situation and encourage improved self-regulatory health habits [59].

The present findings also demonstrated that several demographic characteristics were significantly associated with post-intervention self-management practice scores. Participants aged over 40 years, married individuals, those with university-level education, and those reporting sufficient or higher income achieved significantly higher self-management scores following the eHealth intervention. Although the present study was not designed to determine the mechanisms underlying these associations, previous evidence suggests that educational attainment, digital competencies, and the availability of social and economic support may influence patients’ engagement with digital health interventions and their ability to adopt effective self-management behaviors. These findings emphasize the importance of considering individual patient characteristics when designing and implementing personalized eHealth interventions for patients with cancer, thereby maximizing patient engagement and improving self-management outcomes [6].

## 6. Limitations

Although the present study demonstrated encouraging findings regarding the effectiveness of the eHealth intervention, several factors should be considered when interpreting the results. The study relied on self-reported measures, which may be subject to response bias despite the use of validated instruments. In addition, the relatively short intervention period and the absence of long-term follow-up limited the evaluation of whether the observed improvements in self-management practices and coping behaviors were maintained over time. Furthermore, participants were recruited from two cities within Riyadh Province, Saudi Arabia; therefore, the findings should be generalized to other populations and healthcare settings with appropriate caution. Finally, this trial was retrospectively registered after the completion of participant recruitment and data collection because of administrative and institutional requirements governing clinical trial registration. The study protocol, intervention procedures, and outcome measures remained unchanged throughout the study; however, prospective trial registration would have further strengthened methodological transparency and is recommended for future research.

## 7. Conclusions

The present randomized controlled trial demonstrated that the eHealth intervention significantly improved self-management practices and coping behaviors among patients with cancer compared with usual care. Furthermore, improvements in coping behaviors were positively associated with better self-management practices following the intervention, highlighting the complementary relationship between psychological adaptation and effective disease self-management. These findings support the effectiveness of digitally delivered educational interventions in enhancing patient engagement and promoting active participation in cancer care.

From a clinical perspective, the findings suggest that integrating theory-driven eHealth interventions into routine oncology practice may strengthen patients’ ability to manage treatment-related challenges while improving psychosocial adaptation. The incorporation of Self-Determination Theory constructs, including autonomy, competence, and relatedness, provides a theoretical explanation for the observed improvements and supports the use of patient-centered digital interventions within comprehensive cancer care. Nevertheless, successful implementation requires consideration of contextual factors such as digital literacy, cultural beliefs, technology accessibility, and healthcare infrastructure.

The findings also have important implications for healthcare practice and policy. Healthcare organizations should consider incorporating personalized eHealth programs into routine oncology services and provide appropriate training for healthcare professionals to facilitate patient engagement with digital platforms. Particular attention should be directed toward patients with lower educational attainment, limited digital literacy, older age, or socioeconomic constraints through simplified interfaces, structured onboarding sessions, technical support, and family-assisted digital care models to maximize accessibility and equitable utilization.

Future research should examine the long-term sustainability of intervention effects through longitudinal follow-up studies and evaluate the effectiveness of eHealth interventions across different cancer types, disease stages, and healthcare settings. Additional studies should also investigate culturally tailored digital interventions and scalable implementation strategies that address variations in digital readiness, thereby supporting broader integration of eHealth technologies into national cancer care programs.

## Figures and Tables

**Table 1:** Summary of Intervention Activities.

Week	Content Focus	Delivery Mode	Duration	Fidelity/Adherence Monitoring
1–2	Introduction, basic self-management & coping strategies	Zoom/Google Meet	2 sessions/week	Attendance, WhatsApp engagement
3–4	Advanced coping strategies, scenario-based discussions	Zoom/Google Meet	2 sessions/week	Attendance, WhatsApp participation
5–6	Review, reinforcement, peer discussions, and material sharing	Zoom/Google Meet	2 sessions/week	Attendance, WhatsApp activity, and assignment completion

**Table 2:** Comparison of the demographic characteristics of the Study Sample (n = 212).

	Study Group (106)		Control Group (106)		Test/*p*-Value		Effect Size	Interpretation
Items	n	%	N	%				
Age							Cramér’s V	Very small
<30	41	38.7	40	37.7				
30–40	37	34.9	37	34.9	Chi–square			
>40	28	26.4	29	27.4	0.030	0.985		
Mean ± SD	34.5 ± 11.7		34.3 ± 10.8		0.152	0.879		
Range	17–65		17–65					
Gender							Cramér’s V	Very small
Male	15	14.2	16	15.1	Chi–square			
Female	91	85.8	90	84.9	0.038	0.846		
Marital Status							Cramér’s V	Very small
Married	47	44.3	50	47.2	Chi–square			
Unmarried	59	55.7	56	52.8	0.171	0.679		
Education							Cramér’s V	Very small
Read and write	3	2.8	5	4.7				
Secondary Education	22	20.8	21	19.8	Fisher’s exact test			
University Education	81	76.4	80	75.5	0.529	0.767		
Occupation							Cramér’s V	Very small
Not working	65	61.3	57	53.8	Chi–square			
Working	41	38.7	49	46.2	1.236	0.266		
Income							Cramér’s V	Small
Not enough	22	20.8	34	32.1				
Enough	74	69.8	60	56.6	Chi–square			
Enough and more	10	9.4	12	11.3	4.216	0.121		
Income Source							Cramér’s V	Small
Retirement Salary	66	62.3	49	46.2				
Children’s Financial Support	4	3.8	8	7.5				
Relatives’ Financial Support	4	3.8	7	6.6				
Social Affairs Financial Support	14	13.2	19	17.9	Fisher’s exact test			
Outside work	18	17.0	23	21.7	6.032	0.197		
Living Status							Cramér’s V	Very small
Alone	16	15.1	20	18.9	Chi–square			
With family	90	84.9	86	81.1	0.535	0.464		
Age (continuous) Independent *t*-test		*t* = 0.152		0.018			Cohen’s d	Very small

Note: Chi-square test was used for most categorical variables. Fisher’s exact test was applied when the expected cell count was <5. The use of these tests is appropriate for comparing categorical variables between two independent groups. Cramér’s V effect size was used for categorical variables based on Chi-square or Fisher’s exact tests. Interpretation thresholds: 0.10 = small, 0.30 = medium, 0.50 = large. Cohen’s d was used for the mean difference in age; values < 0.20 indicate a trivial effect. All effect sizes indicate negligible practical differences between the study and control groups at baseline.

**Table 3:** Distribution of Participants by Cancer Type.

	Study Group		Control Group		Test/*p*-Value	
Items	n	%	n	%		
Type of Cancer						
Cervical Cancer	9	8.5	11	10.4		
Colon Cancer	16	15.1	20	18.9		
Blood Cancer	9	8.5	19	17.9		
Breast Cancer	36	34.0	21	19.8		
Prostate Cancer	14	13.2	17	16.0	Fisher’s exact test	
Other	22	20.8	18	17.0	8.854	0.115
Cancer stage						
1st	35	33.0	29	27.4		
2nd	44	41.5	37	34.9		
3rd	22	20.8	30	28.3	Chi–square	
4th	5	4.7	10	9.4	4.065	0.255

Note: Chi-square test was used for most categorical variables. Fisher’s exact test was applied when the expected cell count was <5. The use of these tests is appropriate for comparing categorical variables between two independent groups.

**Table 4:** Differences between Participants’ Self-Management Practices Mean Scores Before and After the Intervention.

Variable Self-Management Practices	Study Group		Paired *t*-Test 1	Control Group		Paired *t*-Test 2	*t*-Test 1	*t*-Test 2
	Pre-Intervention	Post-Intervention		Pre-Intervention	Post-Intervention			
	Mean ± SD	Mean ± SD		Mean ± SD	Mean ± SD			
Acquiring problem-solving (AP)	35.3 ± 15.8	41.0 ± 18.0	*t* = 2.450, *p* = 0.015 *	33.8 ± 15.5	35.6 ± 16.2	*t* = 0.826, *p* = 0.409	*t* = 0.697, *p* = 0.486	*t* = 2.295, *p* = 0.022 *
Managing chemotherapy-related symptoms (MC)	61.9 ± 25.8	74.3 ± 32.5	*t* = 3.076, *p* = 0.002 *	61.0 ± 21.6	64.4 ± 22.4	*t* = 1.124, *p* = 0.261	*t* = 0.260 *p* = 0.795	*t* = 2.604, *p* = 0.010 *
Managing emotional and interpersonal disturbance (ME)	21.1 ± 10.5	24.3 ± 11.2	*t* = 2.146, *p* = 0.033 *	20.6 ± 10.1	21.2 ± 9.7	*t* = 0.441, *p* = 0.659	*t* = 0.353, *p* = 0.724	*t* = 2.154, *p* = 0.032 *
Self-management Score	118.2 ± 50.6	139.7 ± 59.3	*t* = 2.839, *p* = 0.005 *	115.5 ± 29.3	121.2 ± 28.7	*t* = 1.430, *p* = 0.154	*t* = 0.485, *p* = 0.628	*t* = 2.891, *p* = 0.004 *

Paired *t*-test 1: Study group (Pre-intervention/Post-intervention). Paired *t*-test 2: Control group (Pre-intervention/Post-intervention). Independent-samples *t*-test 1: Study vs. Control at baseline. Independent-samples *t*-test 2: Study vs. Control post-intervention. * *p* < 0.05.

**Table 5:** Differences between Participants’ Coping Behaviors Mean Scores Before and After the Intervention.

Variable Coping Behaviors Scores	Study Group		Paired *t*-Test 1	Control Group		Paired *t*-Test 2	*t*-Test 1	*t*-Test 2
	Pre-Intervention	Post-Intervention		Pre-Intervention	Post-Intervention			
	Mean ± SD	Mean ± SD		Mean ± SD	Mean ± SD			
Affective–Oriented Behaviors	40.4 ± 14.1	44.5 ± 12.4	*t* = 2.248, *p* = 0.025 *	39.6 ± 13.4	40.2 ± 15.6	*t* = 0.300, *p* = 0.764	*t* = 0.423, *p* = 0.672	*t* = 2.221, *p* = 0.027 *
Problem–Oriented Coping Behaviors	25.7 ± 10.1	29.4 ± 9.7	*t* = 2.720, *p* = 0.007 *	25.3 ± 9.1	26.5 ± 10.0	*t* = 0.913, *p* = 0.361	*t* = 0.302, *p* = 0.762	*t* = 2.143, *p* = 0.033 *
Jaloweic Coping Total Score	66.1 ± 21.2	73.9 ± 19.3	*t* = 2.801, *p* = 0.005 *	65.0 ± 15.6	66.7 ± 19.2	*t* = 0.707, *p* = 0.480	*t* = 0.430, *p* = 0.667	*t* = 2.722, *p* = 0.007 *

Paired *t*-test 1: Study group (Pre-intervention/Post-intervention). Paired *t*-test 2: Control group (Pre-intervention/Post-intervention). Independent-samples *t*-test 1: Study vs. Control at baseline. Independent-samples *t*-test 2: Study vs. Control post-intervention. * *p* < 0.05.

**Table 6:** Correlation between Coping Behaviors and Self-Management Practices Scores.

Variable	Self-Management Practices			
	Study Group		Control Group	
Coping Behaviors	R	*p*	r	*p*
Pre–intervention	0.091	0.351	0.048	0.622
Post–intervention	0.267	0.006 **	0.081	0.412

Note. R = Pearson correlation coefficient; *p*-values are two-tailed. ** *p*< 0.01.

**Table 7:** (**A**) Association between Participants’ Demographic Characteristics and Coping Behaviors’ Mean Scores for the study group. (**B**) Association between Participants’ Demographic Characteristics and Coping Behaviors’ Mean Scores for the control group.

**(A)**				
	**Pre-Intervention**	**Post-Intervention**	**In-Between Group Comparison**	
	**Mean ± SD**	**Mean ± SD**	**Post Hoc Test**	***p***
Education				
Read and write	70.3 ± 8.6	50.3 ± 24.3	Read and write the Secondary Education	0.155
Secondary Education	67.5 ± 24.7	72.7 ± 24.8	Read and write vs. University Education	0.131
University Education	68.9 ± 23.5	81.2 ± 24.2	Secondary Education vs. University Education	0.032 *
One-way ANOVA [F, *p*]	F = 0.035, *p* = 0.966	F = 3.139, *p* = 0.047 *		
Income				
Not enough	75.4 ± 25.6	60.6 ± 23.7	Not enough vs. Enough	0.026 *
Enough	65.4 ± 21.8	74.1 ± 24.9	Not enough vs. Enough and more	0.028 *
Enough and more	78.0 ± 25.9	80.8 ± 21.1	Enough vs. Enough and more	0.419
One-way ANOVA [F, *p*]	F = 2.521, *p* = 0.085	F = 3.339, *p* = 0.039 *		
**(B)**				
	**Pre-Intervention**	**Post-Intervention**	**In-Between Group Comparison**	
	**Mean ± SD**	**Mean ± SD**	**Post Hoc Test**	***p***
Education				
Read and write	70.2 ± 5.9	67.4 ± 13.3	Read and write vs. Secondary Education	0.684
Secondary Education	63.7 ± 12.5	64.4 ± 20.4	Read and write vs. University Education	0.750
University Education	65.0 ±16.7	68.6 ± 19.2	Secondary Education vs. University Education	0.651
One-way ANOVA [F, *p*]	F = 0.346, *t* = 0.708	F = 0.393, *t* = 0.676		
Income				
Not enough	61.4 ± 12.0	65.7 ± 19.8	Not enough vs. Enough	0.226
Enough	67.0 ± 16.9	69.7 ± 18.9	Not enough vs. Enough and more	0.774
Enough and more	65.0 ± 17.1	63.8 ± 19.2	Enough vs. Enough and more	0.593
One-way ANOVA [F, *p*]	F = 1.377, *t* = 0.257	F = 0.763, *t* = 0.469		

* *p* < 0.05.

**Table 8:** (**A**) Association between Participants’ Demographic Characteristics and Mean Scores of Self-Management Practices for the study group. (**B**) Association between Participants’ Demographic Characteristics and Mean Scores of Self-Management Practices for the control group.

**(A)**				
	**Pre-Intervention**	**Post-Intervention**	**In-Between Group Comparison**	
	**Mean ± SD**	**Mean ± SD**	**Post Hoc Test**	***p***
Age				
<30	111.9 ± 47.8	110.3 ± 52.9	<30 vs. 30–40	0.005 *
30–40	118.5 ± 54.0	150.4 ± 58.3	30–40 vs. >40	0.378
>40	118.2 ± 50.6	168.6 ± 51.7	<30 vs. > 40	<0.001 **
One-way ANOVA [F, *p*]	F = 0.204, *p* = 0.815	F = 10.595, *p* < 0.001 **		
Marital Status				
Single	110.9 ± 52.9	111.6 ± 56.7	Single vs. Married	<0.001 **
Married	121.7 ± 48.1	163.6 ± 51.9	Single vs. Divorced	0.082
Divorced	128.7 ± 51.8	147.0 ± 56.7	Married vs. Divorced	0.562
One-way ANOVA [F, *p*]	F = 0.880, *p* = 0.418	F = 10.462, *p* < 0.001 ***		
Education				
Read and write	151.3 ± 35.6	108.0 ± 37.4	Read and write vs. Secondary Education	0.613
Secondary Education	118.6 ± 47.8	124.4 ± 53.2	Read and write vs. University Education	0.104
University Education	119.2 ± 51.6	166.9 ± 61.4	Secondary Education vs. University Education	0.004 *
One-way ANOVA [F, *p*]	F = 0.592, *p* = 0.554	F = 5.447, *p* = 0.006 **		
Income				
Not enough	102.7 ± 44.1	105.5 ± 60.5	Not enough vs. Enough	0.053
Enough	124.3 ± 51.8	131.3 ± 62.3	Not enough vs. Enough and more	0.014 *
Enough and more	107.1 ± 49.9	165.0 ± 57.2	Enough vs. Enough and more	0.109
One-way ANOVA [F, *p*]	F = 1.839, *p* = 0.164	F = 3.370, *p* = 0.038 *		
Income Source				
Retirement Money	118.4 ± 50.7	143.6 ± 62.9	Retirement Money vs. Sons’ help	0.152
Sons’ help	114.0 ± 51.5	211.0 ± 10.4	Retirement Money vs. Relatives	0.953
Relatives	111.5 ± 44.6	164.5 ± 49.1	Retirement Money vs. Social Help	0.992
Social Affairs	111.0 ± 52.9	136.1 ± 34.4	Retirement Money vs. Outside work	0.115
Outside work	108.9 ± 31.3	106.8 ± 51.9	Sons’ help vs. Relatives	0.775
One-way ANOVA [F, *p*]	F = 0.446, *p* = 0.774	F = 3.367, *p* = 0.012 *	Sons ’s help vs. Social Help	0.146
			Sons ’s help vs. Outside work	0.011 *
			Relatives vs. Social Help	0.903
			Relatives vs. Outside work	0.358
			Social Help vs. Outside work	0.598
**(B)**				
	**Pre-Intervention**	**Post-Intervention**	**In-Between Group Comparison**	
	**Mean ± SD**	**Mean ± SD**	**Post Hoc Test**	***p***
Age				
<30	114.2 ± 30.0	120.6 ± 30.1	<30 vs. 30–40	0.658
30–40	120.1 ± 27.2	121.6 ± 26.6	30–40 vs. >40	0.911
>40	111.3 ± 31.2	125.2 ± 30.0	<30 vs. > 40	0.451
One-way ANOVA [F, *p*]	F = 0.787, *t* = 0.458	F = 0.225, *t* = 0.799		
Marital Status				
Single	115.3 ± 27.4	123.1 ± 31.3	Single vs. Married	0.984
Married	114.7 ± 31.1	124.1 ± 26.1	Single vs. Divorced	0.291
Divorced	119.6 ± 31.6	108.1 ± 27.5	Married vs. Divorced	0.242
One-way ANOVA [F, *p*]	F = 0.113, *t* = 0.894	F = 1.357, *t* = 0.262		
Education				
Read and write	128.2 ± 32.9	115.0 ± 23.6	Read and write vs. Secondary Education	0.266
Secondary Education	105.6 ± 26.3	119.2 ± 31.0	Read and write vs. University Education	0.694
University Education	117.3 ± 29.5	123.4 ± 28.6	Secondary Education vs. University Education	0.234
One-way ANOVA [F, *p*]	F = 1.848, *t* = 0.163	F = 0.343, *t* = 0.710		
Income				
Not enough	115.4 ± 30.8	111.3 ± 25.2	Not enough vs. Enough	0.895
Enough	112.6 ± 29.8	126.2 ± 29.3	Not enough vs. Enough and more	0.051
Enough and more	130.1 ± 17.6	133.4 ± 27.3	Enough vs. Enough and more	0.143
One-way ANOVA [F, *p*]	F = 1.810, *t* = 0.169	F = 4.199, *t* = 0.018		
Income Source				
Retirement Money	113.1 ± 26.4	126.1 ± 31.4	Retirement Money vs. Sons’ help	0.989
Sons’ help	122.0 ± 29.4	120.8 ± 27.0	Retirement Money vs. Relatives	0.714
Relatives	115.9 ± 28.6	111.3 ± 19.9	Retirement Money vs. Social Help	0.960
Social Affairs	118.7 ± 32.2	120.7 ± 25.3	Retirement Money vs. Outside work	0.870
Outside work	115.5 ± 34.5	119.0 ± 28.6	Sons’ help vs. Relatives	0.970
One-way ANOVA [F, *p*]	F = 0.232, *t* = 0.920	F = 0.555, *t* = 0.696	Sons ’s help vs. Social Help	0.947
			Sons ’s help vs. Outside work	0.972
			Relatives vs. Social Help	0.870
			Relatives vs. Outside work	0.947
			Social Help vs. Outside work	0.714

* *p* < 0.05, ** *p* < 0.01, *** *p* < 0.001.

## Data Availability

The data presented in this study are available from the corresponding author upon reasonable request. The data are not publicly available because they contain information that could compromise the privacy of the participants and are subject to ethical.
